# Surface functionalization-specific binding of coagulation factors by zinc oxide nanoparticles delays coagulation time and reduces thrombin generation potential *in vitro*

**DOI:** 10.1371/journal.pone.0181634

**Published:** 2017-07-19

**Authors:** Jun-Young Yang, Jiyeong Bae, Ayoung Jung, Seonyeong Park, Seungtae Chung, Jihyun Seok, Hangsik Roh, Youngju Han, Jae-Min Oh, Soojung Sohn, Jayoung Jeong, Wan-Seob Cho

**Affiliations:** 1 Toxicological Research Division, National Institute of Food and Drug Safety Evaluation, Ministry of Food and Drug Safety, Osong, Republic of Korea; 2 Department of Medicinal Biotechnology, School of Health Sciences, Dong-A University, Busan, Republic of Korea; 3 Department of Chemistry and Medical Chemistry, College of Science and Technology, Yonsei University, Gangwon-do, Republic of Korea; Institute of Materials Science, GERMANY

## Abstract

Zinc oxide nanoparticles (ZnO NPs) have many biomedical applications such as chemotherapy agents, vaccine adjuvants, and biosensors but its hemocompatibility is still poorly understood, especially in the event of direct contact of NPs with blood components. Here, we investigated the impact of size and surface functional groups on the platelet homeostasis. ZnO NPs were synthesized in two different sizes (20 and 100 nm) and with three different functional surface groups (pristine, citrate, and L-serine). ZnO NPs were incubated with plasma collected from healthy rats to evaluate the coagulation time, kinetics of thrombin generation, and profile of levels of coagulation factors in the supernatant and coronated onto the ZnO NPs. Measurements of plasma coagulation time showed that all types of ZnO NPs prolonged both active partial thromboplastin time and prothrombin time in a dose-dependent manner but there was no size- or surface functionalization-specific pattern. The kinetics data of thrombin generation showed that ZnO NPs reduced the thrombin generation potential with functionalization-specificity in the order of pristine > citrate > L-serine but there was no size-specificity. The profile of levels of coagulation factors in the supernatant and coronated onto the ZnO NPs after incubation of platelet-poor plasma with ZnO NPs showed that ZnO NPs reduced the levels of coagulation factors in the supernatant with functionalization-specificity. Interestingly, the pattern of coagulation factors in the supernatant was consistent with the levels of coagulation factors adsorbed onto the NPs, which might imply that ZnO NPs simply adsorb coagulation factors rather than stimulating these factors. The reduced levels of coagulation factors in the supernatant were consistent with the delayed coagulation time and reduced potential for thrombin generation, which imply that the adsorbed coagulation factors are not functional.

## Introduction

The rapid increase in the need and usage of nanomaterials in various fields may necessarily increase human exposure to these materials, but the potential hazards and interactions with biological systems are still poorly understood [1, 2]. Recently, nanomaterials have been applied in biomedical applications such as drug delivery vehicles, therapeutic agents, biosensors, and laboratory diagnostics and zinc oxide nanoparticles (ZnO NP) are one of the leading candidates for these applications [3]. Various types of ZnO can be synthesized not only by controlling the size but also by modifying the shape, including spherical-, fiber-, whisker-, and tetrapod-shapes. Spherical ZnO NPs are the most popular type, which can be used for various applications including as anti-cancer and anti-bacterial agents, and one of the main mechanisms for these applications is production of reactive oxygen species (ROS) and induction of apoptosis [4–6]. Tetrapod-shaped ZnO NPs were recently studied for antibacterial activity [7], as antiviral agents (for example, herpes simplex virus type-1 and type-2) [8–10], and as a vaccine adjuvant [8] because of their unique three-dimensional geometry. In addition, composite types of nanostructures are also synthesized in various forms including ZnO quantum dots and ZnO nanoclusters for the purpose of anti-cancer activity as well as anti-bacterial agents [11–13]. Because of the various applications and advantages of ZnO NPs, the Organization for Economic Cooperation and Development (OECD) initiative adopted ZnO NP as one of 13 nanomaterials for evaluation [14].

Among various administration routes, intravenous injection can be applied for drug delivery, imaging, and sensors but this route of administration has one of the highest risks, which warrants precise and thorough studies [15, 16]. Intravenously injected NPs can contact directly with blood components, including platelets. Thus, disorders of platelets and coagulation factors by NPs can result life-threatening situations by increasing or decreasing blood clotting: activation of platelets by NPs can produce platelet aggregation and thrombus formation, while suppression of platelets by NPs can produce spontaneous and excessive bleeding [17]. There are several reports regarding platelet homeostasis by NPs but the results showed discrepancies between NPs. For instance, silver NPs incubated with plasma showed activation of platelet aggregation in some studies [18–20], and inhibition of platelet aggregation in others [21, 22].

In addition to platelet aggregation, the generation of thrombin by NPs can produce fatal situations such as disseminated intravascular coagulation and atherosclerosis. Therefore, the rapid assessment of NPs regarding platelet homeostasis is important in the development of hemo-compatible NPs. The size and surface properties of NPs are generally recognized as major physicochemical parameters determining their toxic potential but there is little information concerning the role of these factors in platelet homeostasis [16]. Therefore, in this study, we evaluated the roles of size and surface functionalization of ZnO NPs on the hematological homeostasis using ZnO NPs having two different sizes (20 nm and 100 nm) and three different surface modifications (pristine, L-serine, and citrate).

## Materials and methods

### Synthesis of ZnO NPs with two different sizes and three different functional groups

In this study, we used two sets (20 nm and 100 nm) of three different functionalized ZnO NPs (pristine, citrate, and L-serine). The pristine 20 nm-sized ZnO NPs were purchased from Sumitomo Osaka Cement Co, Ltd. (Lot#: 141319; Tokyo, Japan) and 100 nm-sized ZnO NPs were obtained from American Elements (Lot#: 1871511079–673; Los Angeles, CA, USA). For the pristine ZnO NPs, surface modification was performed to provide negative or positive zeta potential according to previously described methods [23]. Briefly, negatively charged ZnO NPs were prepared by incubating pristine ZnO NPs with citrate/4-(2-hydroxyethyl)-1-piperazineethanesulfonic acid (HEPES) buffer solution at 20% wt/v ratio for 1 min. Citrate/HEPES buffer solution was prepared by dissolving sodium citrate (Sigma-Aldrich, St Louis, MO, USA) in 20 mM HEPES (Sigma-Aldrich) to 1% wt/v ratio at pH 7.0. Positively charged ZnO NPs were prepared by incubating pristine ZnO NP with L-serine/HEPES buffer solution at 20% wt/v ratio for 1 min. L-serine/HEPES buffer solution was prepared by dissolving L-serine in 20 mM HEPES (Sigma-Aldrich) to 1% wt/v ratio at pH 6.0.

### Physicochemical characterization of ZnO NPs

The primary size and morphology of the ZnO NPs was analyzed by scanning electron microscopy (SEM) imaging using a FEI Quanta 250 FEG instrument (Hillsboro, OR, USA). The ZnO samples were diluted in deionized water (DW), and a drop of suspension was loaded onto a Si wafer. The specimens were dried in air and subjected to microscopic study. The crystal structure of all ZnO samples was characterized using an X-ray diffractometer (XRD) employing a Bruker D2PHASER with Ni-filtered CuKα radiation (λ = 1.5406 Å). The zeta potential and hydrodynamic size was measured using an ELSZ-1000 instrument (Otsuka Electronics Co, Ltd, Osaka, Japan). Briefly, ZnO NPs were dispersed in DW at a concentration of ~10 μg/mL and were vigorously stirred at 25°C for 1 hr. All measurements were carried out three times using a zeta flow cell (Otsuka Electronics Co, Ltd) or a disposable dynamic light scattering cuvette (Ratiolab GmbH, Dreieich, Germany) for zeta potential and hydrodynamic radius measurements, respectively. To confirm the presence of citrate or L-serine on the surface of ZnO NPs, Fourier transform infrared (FT-IR) spectra of the samples were obtained on a Spectrum One FT-IR spectrometer (Perkin-Elmer, Downers Grove, Il, USA), using the conventional KBr pellet method [24]. Typically, 1 part sample was ground with 200 parts of KBr in agate mortar then a transparent pellet was prepared with a hand press. FT-IR spectra were obtained in the wavenumber range of 4,000 ~ 400 cm^-1^ with resolution 16 cm^-1^.

### Collection of blood from Wistar rats

Six-week-old specific-pathogen free male Wistar rats were purchased from Orient Bio (Gyeonggi, Korea) and acclimatized for 1 week before experiments. The protocol for animal experiments was approved by the Animal Care and Use Committee of the National Institute of Food and Drug Safety (NIFDS). All animal experiments were performed in the facility accredited by the Association for Assessment and Accreditation of Laboratory Animal Care (AAALAC). Rats were euthanized by intraperitoneal injection of tiletamine-zolazepam (Zoletil^®^) (Virbac, Fort Worth, TX, USA) at 50 mg/kg body weight and whole blood was collected via the abdominal aorta using a vacutainer tube containing sodium citrate (BD Diagnostics, Franklin Lakes, NJ, USA).

### Analysis of coagulation time

The plasma samples were incubated with different types of ZnO NPs to determine their effects on blood coagulation. The analysis of coagulation time was performed with slight modification of the ITA-12 protocol that was provided by the Nanotechnology Characterization Laboratory [25]. Briefly, platelet-poor plasma (PPP) was prepared by centrifugation of whole blood at 2500 × *g* for 10 min. Then, 100 μL of ZnO NPs were dispersed in 900 μL of PPP and incubated for 30 min at 37°C. The suspension was then centrifuged at 18000 × *g* for 5 min to collect NP-free supernatants. Then, active partial thromboplastin time (aPTT) and prothrombin time (PT) were measured using the ACL 200 coagulation analyzer (Instrumentation Laboratory, Bedford, MA, USA).

### Thrombin generation assay

To evaluate the effects of NPs on the generation of thrombin, we used a thrombin generation assay (TGA) kit (Technothrombin TGA kit; Technoclone, Vienna, Austria). The thrombin generation assay was performed in the absence of tissue factors to study the effects of intrinsic pathway activation by the NPs [26]. The PPP was obtained by centrifugation of whole blood at 2500 × *g* for 10 min. Then, NPs diluted in HEPES-buffered saline [10 mM HEPES and 150 mM sodium chloride (NaCl, pH 7.4)] were incubated with an equal volume of PPP for 30 min at 37°C. The final concentration of ZnO NPs for TGA was 0.1, 0.25, and 0.5 mg/mL. After incubation, the solution was centrifuged at 15000 × *g* for 10 min to separate the NPs from the remaining supernatant. Then, 40 μL of NP-free supernatant was combined with 50 μL of the TGA substrate (1 mM Z-Gly-Gly-Arg-AMC with calcium chloride, CaCl_2_) and 10 μL of Reagent C (RC) Low, and the fluorescence was measured at 360/460 nm immediately after addition of the substrate. To evaluate the kinetics, the fluorescence measurements were performed at 37°C for 60 min with 1 min intervals using a plate reader (Tecan, Zurich, Switzerland). Thrombin generation was calculated using TGA evaluation software (Technoclone) and the results are given in nM thrombin generated in the sample for each time point.

### Measurement of the levels of coagulation factors

To evaluate the role of size and surface functionalization of ZnO NPs on the activation of coagulation factors and adsorption specificity, ZnO NPs were incubated with PPP and several coagulation factors were measured in the supernatant and in the protein adsorbed onto the surface of ZnO NPs by enzyme linked immunosorbent assay (ELISA) and western blot analysis, respectively. Briefly, ZnO NP (0.5 mg/mL) was incubated with PPP for 30 min at 37°C and NP-free supernatant was collected by centrifugation at 15000 × *g* for 10 min. Then the levels of activated coagulation factors including factors II, III, V, VII, VIII, IX, XI, and XII were measured in the NP-free supernatant using commercial ELISA kits (all kits were purchased from Antibodies-online; Atlanta, GA, USA).

To evaluate the coagulation factors adsorbed onto the surface of ZnO NPs, NP pellets were washed three times with PBS by centrifugation at 15000 × *g* for 10 min. Then, the adsorbed protein was resolved using a 12% sodium dodecyl sulfate-polyacrylamide gel (Bio-Rad, Berkeley, CA, USA) and electro-transferred onto polyvinylidene fluoride membranes (Bio-Rad). The blotted membranes were blocked for 1 hr at 20°C using 5% skim milk in Tris-buffered saline and 0.1% Tween 20. The blocking solution was removed and membranes were incubated overnight with the appropriate dilution of the respective primary antibody: coagulation factors II, III, V, XII, and XIII (Antibodies-online; Atlanta, GA, USA), coagulation factor VII (Abcam; Cambridge, MA, USA), coagulation factor VIII (Santa Cruz Biotechnology; Santa Cruz, CA, USA), and coagulation factors IX and XI (Bioss Inc.; Woburn, MA, USA). Then, the membranes were further incubated with horseradish peroxidase-conjugated secondary antibodies (Cell Signaling Technology; Danvers, MA, USA) specific for the primary antibodies. The membrane was developed using the enhanced chemiluminescence prime western blotting detection reagent (GE Healthcare Life Science; Buckinghamshire, UK) and analysis was performed using ChemiDoc MP System (Bio-Rad).

### Statistical analysis

Data were expressed as means ± SEM. To evaluate statistical significance, one-way analysis of variance (ANOVA) was applied. When the ANOVA test showed statistical significance (*p* < 0.05), each group was compared using post-hoc Tukey’s pairwise comparisons. All statistical analysis and graphs were prepared using GraphPad Prism (version 6.0 for Windows; GraphPad Software, San Diego, CA, USA). A value of *p* < 0.05 was considered statistically significant.

## Results

### Evaluation of size and crystallinity of ZnO NPs

**Fig 1:**
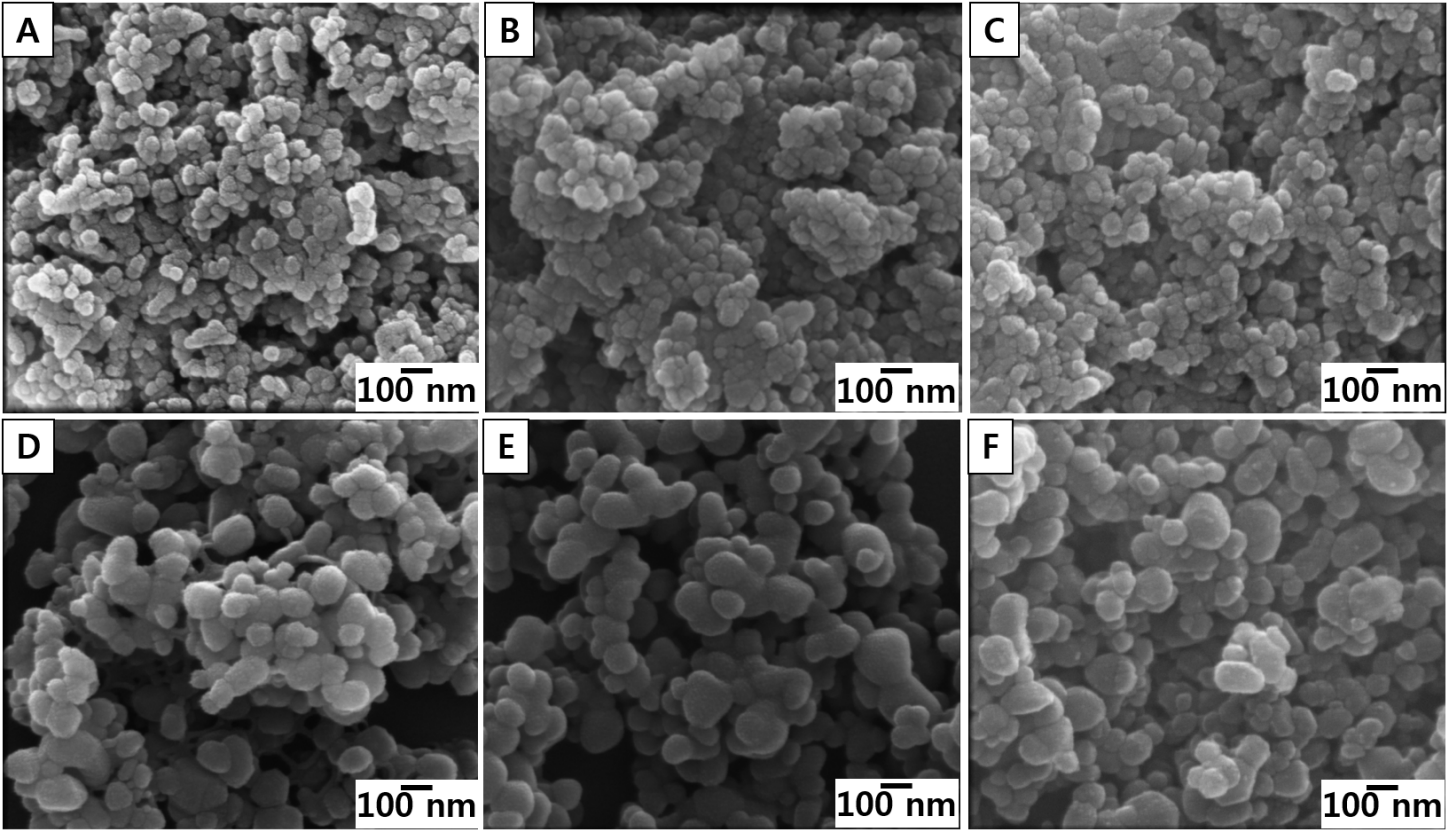
Scanning electron microscopy (SEM) images of ZnO NPs. (A) Pristine 20 nm, (B) Citrate 20 nm, (C) L-serine 20 nm, (D) Pristine 100 nm, (E) Citrate 100 nm, and (F) L-serine 100 nm.

To evaluate the impact of size on hemo-compatibility, 20 nm- and 100 nm-sized ZnO NPs were synthesized. SEM images showed that all types of ZnO NPs were spherically shaped with a narrow size distribution range (Fig 1). Measurements of primary sizes showed that three types of 20 nm-sized ZnO NPs are approximately 26–27 nm and three types of 100 nm-sized ZnO NPs are about 95–100 nm (Table 1). Hydrodynamic size showed that all types of ZnO NPs were slightly agglomerated in DW ranging from approximately 220–400 nm (Table 1). The hydrodynamic size of 20 nm ZnO NPs was similar to that of 100 nm-sized ZnO NPs. The lattice parameters calculated from the 2θ values of ZnO NPs were *a* = 3.251 Å and *c* = 5.206 Å (S1 Fig). These values were consistent with that of a reference compound, wurtzite parameters (*a* = 3.249 Å and *c* = 5.207 Å).

**Table 1:** Physicochemical properties of ZnO NP.

Measurements	20 nm			100 nm				
Modification	Pristine	Citrate	L-serine	Pristine	Citrate		L-serine
Primary size (nm)^a^	26.8 ± 4.5	26.9 ± 4.3	27.1 ± 7.5	95.1 ± 12.9		100.2 ± 22.9	95.3 ± 23	
Hydrodynamic size (nm)^b^	399 ± 16	341 ± 61	219 ± 3	354 ± 11		299 ± 9	384 ± 6	
Zeta potential (mV)	21.4 ± 4.6	-36.0 ± 2.6	30.0 ± 3.4	15.8 ± 1.7		-25.3 ± 57.6	25.4 ± 2.5	

^a^The primary size was measured by SEM.

^b^The hydrodynamic size and zeta potential was measured in DW.

### Zeta potential and FT-IR analysis of ZnO NPs

**Fig 2:**
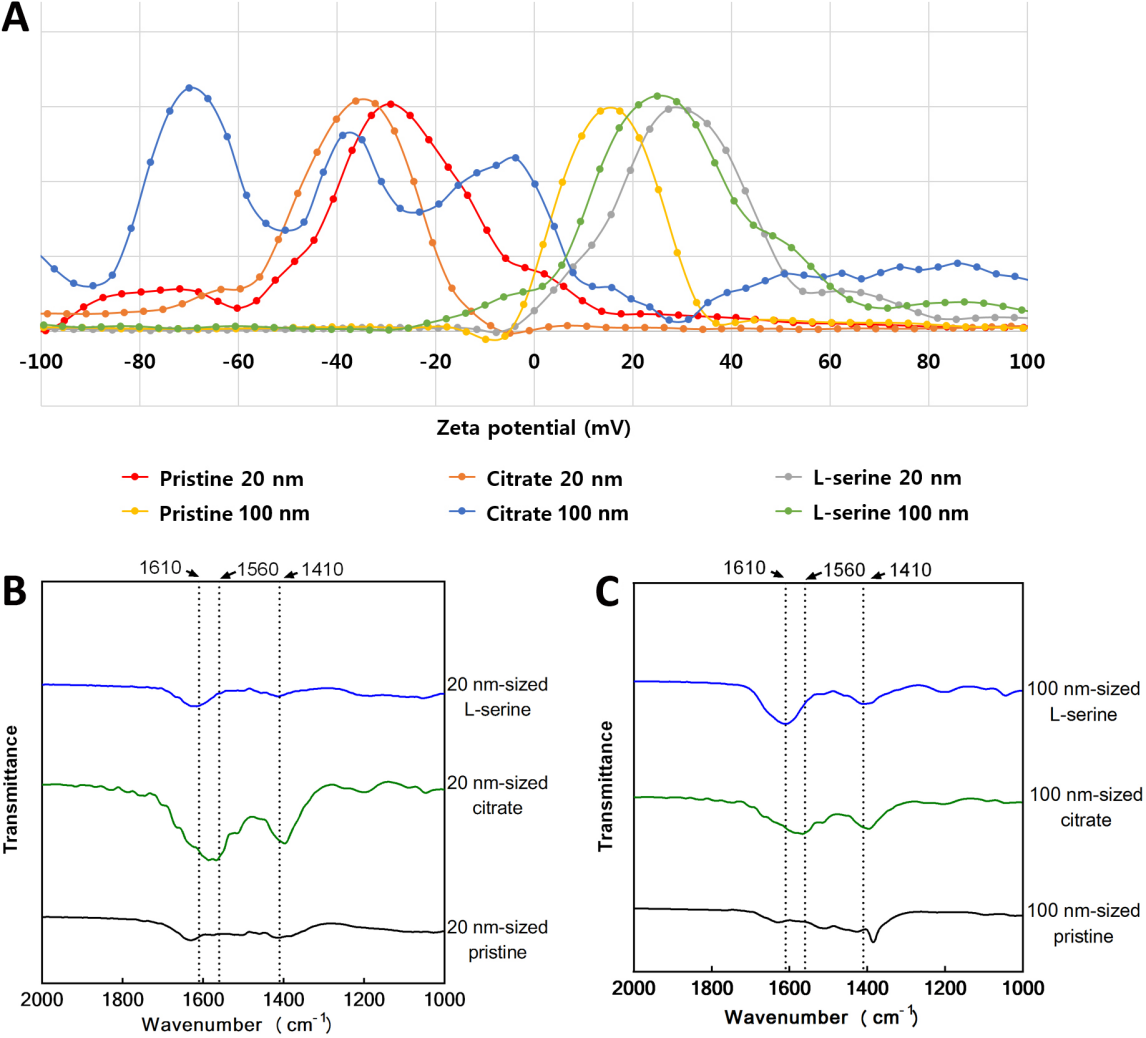
Surface charge and FT-IR analysis of ZnO NPs. **(A) Surface charge of ZnO NPs evaluated by the zeta potential measurement.** FT-IR analysis of (B) 20 nm-sized ZnO NPs and (C) 100 nm-sized ZnO NPs. Note that citrate coated 20 nm- and 100 nm-ZnO NPs showed 1560 and 1410 cm^-1^ that are specific peak for asymmetric COO^-^ and symmetric COO^-^, respectively. While L-serine coated 20 nm- and 100 nm-ZnO NPs showed 1610 and 1410 cm^-1^ which are specific for NH_2_ bending and symmetric COO^-^, respectively.

To evaluate the role of surface functional groups or surface charge on hemo-compatibility, ZnO NPs were conjugated with none (pristine), citrate, or L-serine to provide three distinctive charged surfaces. Zeta potential analysis showed that 20 nm- and 100 nm-sized pristine or L-serine modified ZnO NPs were positively charged, while citrate coated 20 nm- and 100 nm-sized ZnO NPs were negatively charged (Table 1). The distribution of zeta potential showed that all types of ZnO NPs showed narrow single peaks except for 100 nm-sized citrate-coated ZnO NPs that exhibited wide distribution with three main peaks (Fig 2A). FT-IR analysis confirmed that the surface of ZnO NPs was fully functionalized with citrate or L-serine (Fig 2B and 2C). The citrate functionalized ZnO NPs showed 1560 and 1410 cm^-1^ which are specific peaks for asymmetric COO^-^ and symmetric COO^-^, respectively, while L-serine functionalized ZnO NPs showed 1610 and 1410 cm^-1^ that are specific for NH_2_ bending and symmetric COO^-^, respectively.

### Delayed plasma coagulation time by ZnO NPs

**Fig 3:**
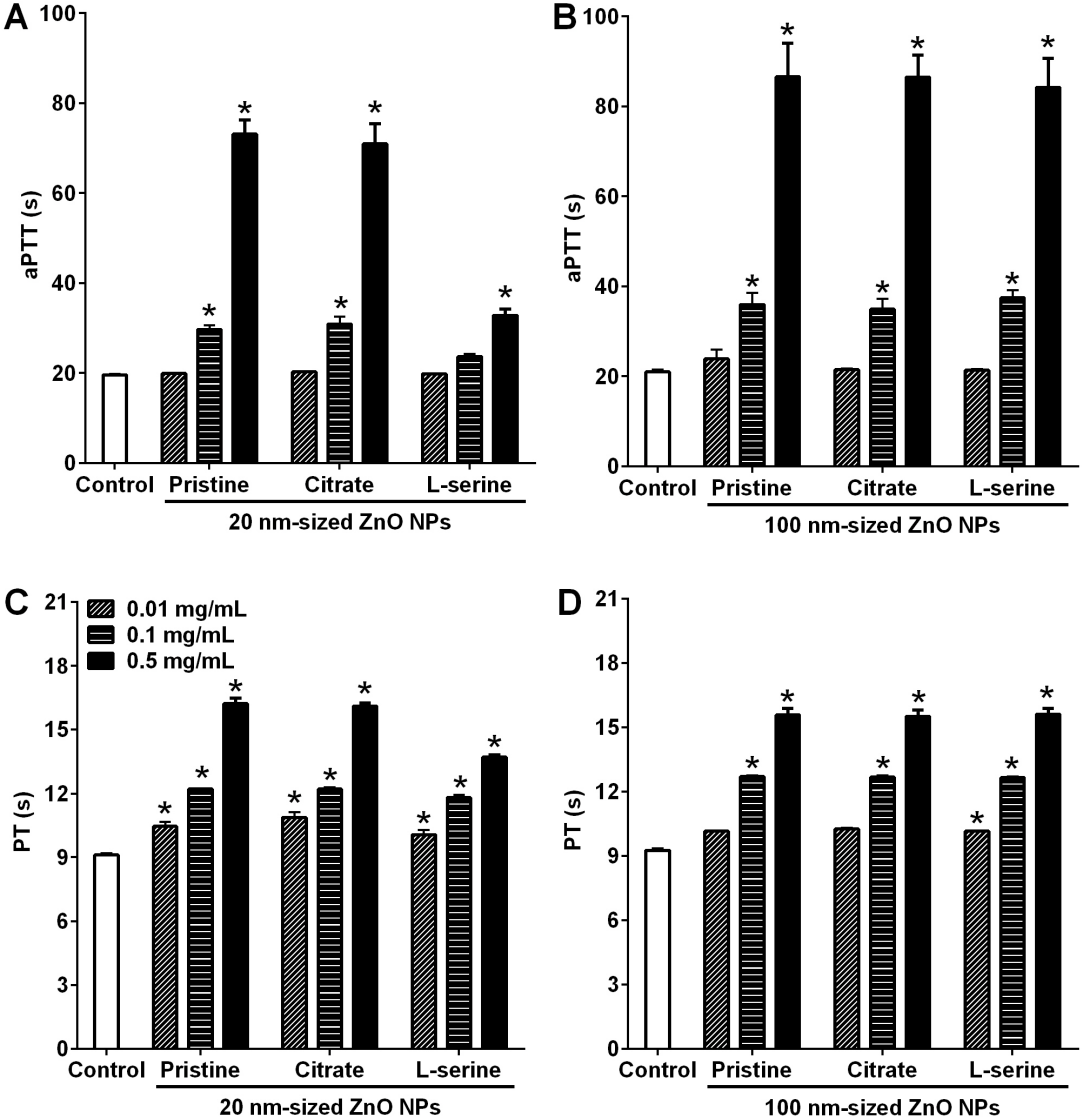
Measurement of active partial thromboplastin time and prothrombin time after incubation of platelet-poor plasma and ZnO NPs. aPTT was measured for (A) 20 nm- and (B) 100 nm-sized ZnO NPs. PT was measured for (C) 20 nm- and (D) 100 nm-sized ZnO NPs. ZnO NPs were conjugated with none (pristine), citrate, and L-serine to provide three distinctive charged surfaces. Each sample was analyzed in duplicate and repeated three times, by using three separate plasma sets. Results are means ± SEM, *p < 0.05 versus control.

The aPTT and PT assays are used to evaluate the role of size or surface functionalization on the intrinsic and extrinsic coagulation times, respectively. All types of ZnO NPs prolonged both aPTT and PT in a dose-dependent manner (Fig 3). However, there was no size- or surface functionalization-dependent pattern in either aPTT or PT.

### Inhibition of thrombin generation by ZnO NPs

**Fig 4:**
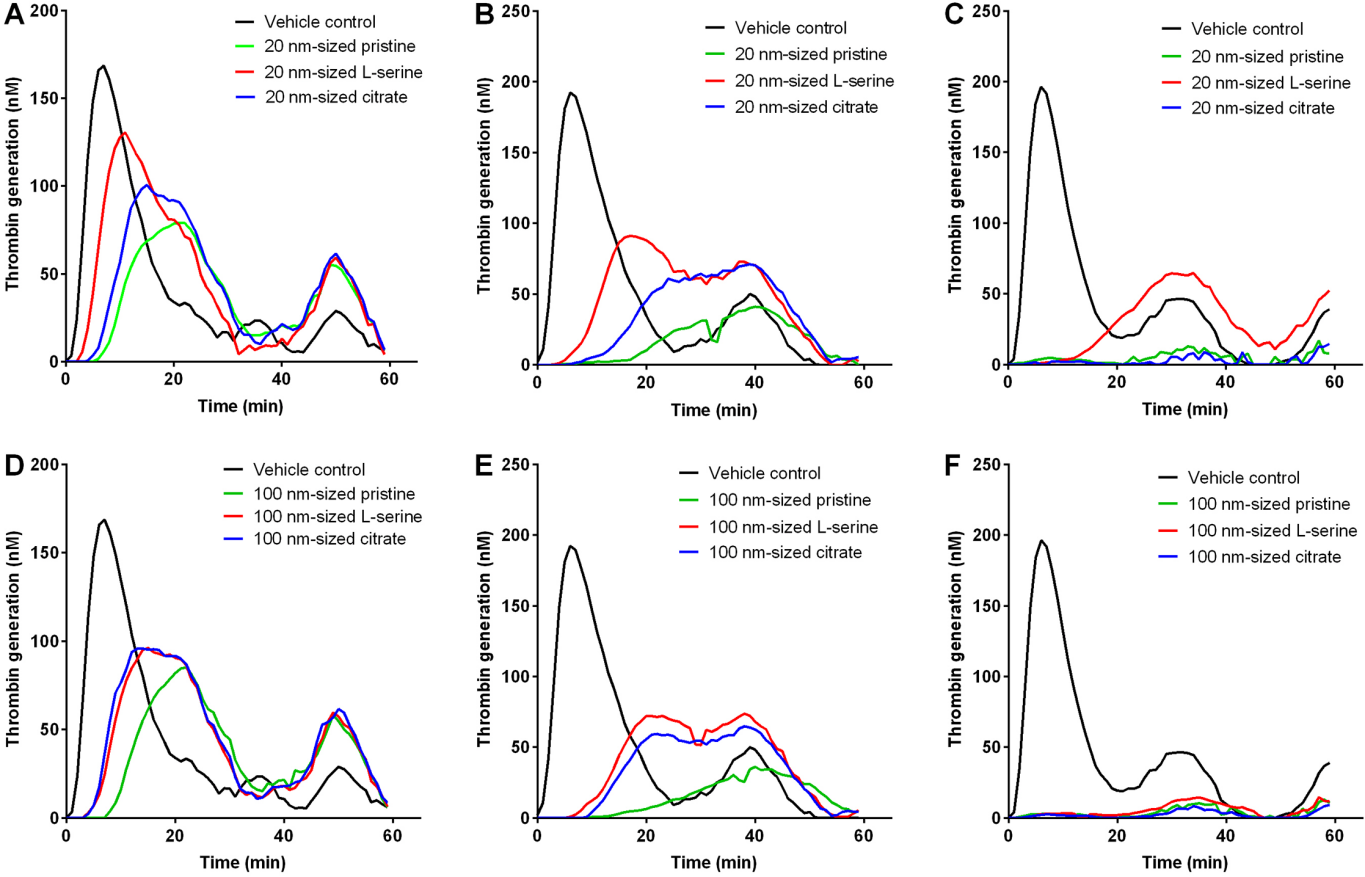
Thrombin generation assay of ZnO NPs in platelet poor plasma. Thrombin generation assay was performed with 20 nm-sized ZnO NPs at (A) 0.1, (B) 0.25, and (C) 0.5 mg/mL. Thrombin generation assay was also performed with 100 nm-sized ZnO NPs at (C) 0.1, (D) 0.25, and (E) 0.5 mg/mL. *n* = 3.

All types of ZnO NPs of both sizes showed less potential for thrombin generation compared to vehicle control in a dose-dependent manner (Fig 4). In addition, the time of onset for thrombin generation was delayed in a dose-dependent manner for all types of ZnO NPs of both sizes. The order of inhibitory potential of thrombin generation for 20 nm- and 100 nm-sized ZnO NP was pristine > citrate > L-serine.

### Profile of the levels of plasma coagulation factors by ZnO NPs

**Fig 5:**
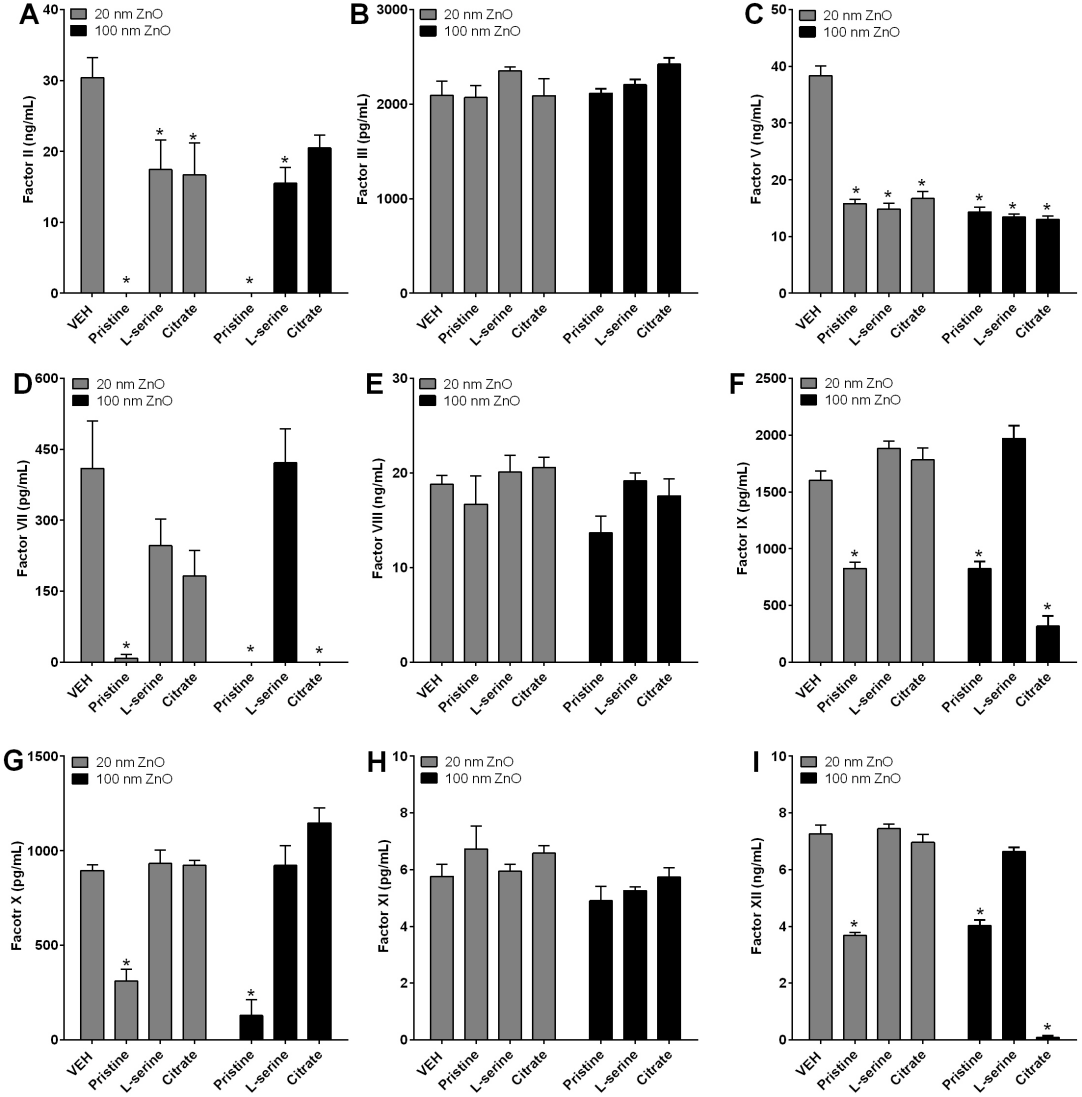
The levels of coagulation factors in the supernatant of platelet poor plasma after incubation with ZnO NPs. **ZnO NPs at 0.5 mg/mL were incubated with PPP and the levels of coagulation factors were measured using ELISA kits in the NP-free supernatant.** (A), factor II; (B), factor III; (C), factor V; (D), factor VII; (E), factor VIII; (F), factor IX; (G), factor X; (H), factor XI; (I), factor XII. *p < 0.05 and *n* = 4.

**Fig 6:**
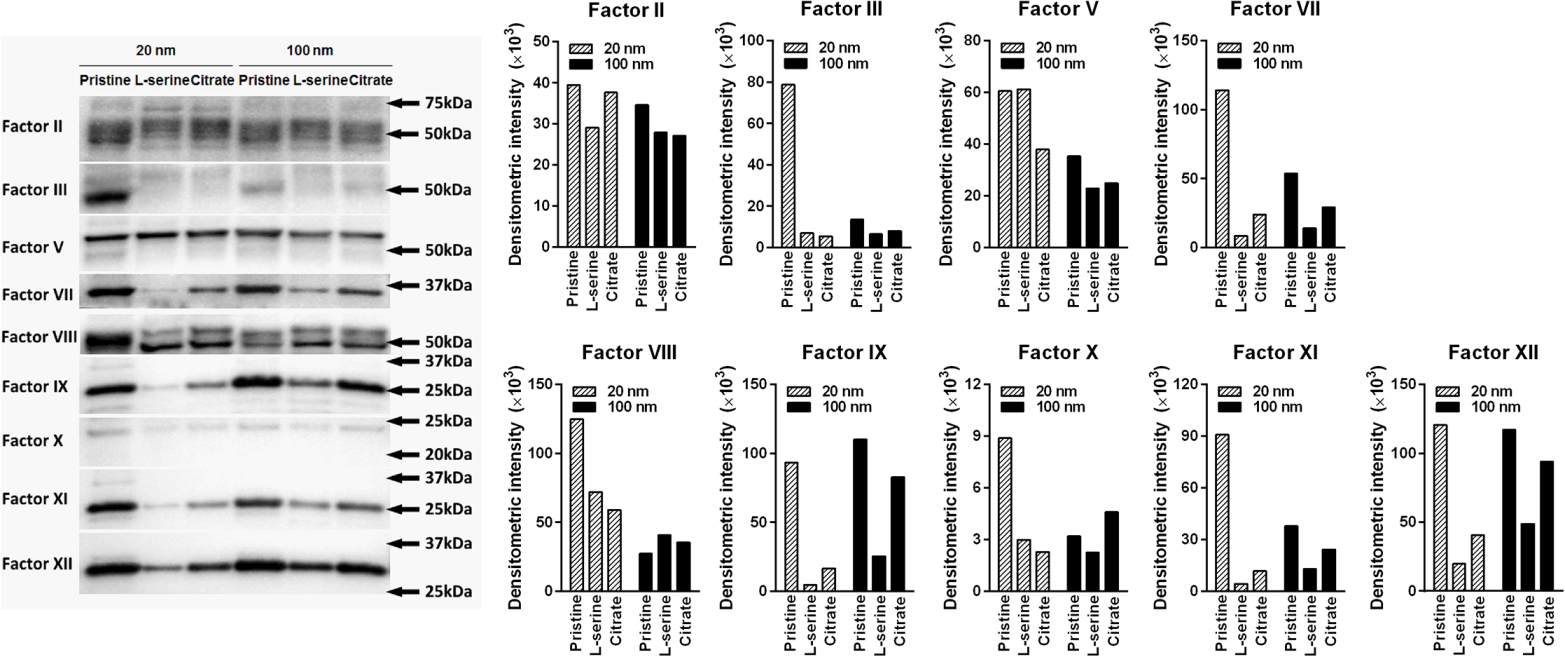
Western blot analysis of ZnO NP-bound coagulation factors on the surface of NPs. ZnO NPs were incubated with PPP and NP pellets were collected and dissolved in loading buffer, then analyzed for coagulation factors using sodium dodecyl sulfate-polyacrylamide gel electrophoresis (SDS-PAGE) followed by immunoblotting. Each factor was separately subjected to densitometric analysis.

To evaluate the profile of coagulation factors activated or depressed by ZnO NPs, PPP was incubated with ZnO NPs and not only the levels of coagulation factors in the supernatant of PPP, but also the levels of NP-bound (coronated) coagulation factors were assayed by ELISA and western blot analysis, respectively. The results of ELISA and western blot analysis are presented in Figs 5 and 6, respectively. Both datasets are also summarized in Table 2.

**Table 2:** Summary of expression profile of coagulation factors observed by ELISA and western blot analysis.

Coagulation factors	Samples	20 nm			100 nm		
		pristine	citrate	L-serine	pristine	citrate	L-serine
Factor II	Supernatant^1^	0%	17%	17%	0%	21%	16%
	Corona^2^	moderate	moderate	mild	moderate	mild	mild
Factor III	Supernatant	99%	100%	112%	101%	115%	105%
	Corona	strong	minimal	minimal	mild	minimal	minimal
Factor V	Supernatant	41%	44%	39%	37%	34%	35%
	Corona	strong	moderate	strong	moderate	mild	mild
Factor VII	Supernatant	2%	44%	60%	0%	0%	103%
	Corona	strong	mild	minimal	strong	strong	mild
Factor VIII	Supernatant	89%	109%	107%	73%	94%	102%
	Corona	strong	strong	strong	mild	moderate	moderate
Factor IX	Supernatant	52%	111%	118%	52%	20%	123%
	Corona	strong	mild	minimal	strong	strong	mild
Factor X	Supernatant	35%	103%	104%	14%	128%	103%
	Corona	minimal	minimal	minimal	minimal	minimal	minimal
Factor XI	Supernatant	117%	103%	114%	85%	100%	91%
	Corona	strong	mild	minimal	moderate	mild	mild
Factor XII	Supernatant	51%	96%	103%	55%	1%	92%
	Corona	strong	moderate	mild	Strong	strong	moderate

^1^The levels of expression were expressed as percentages compared to vehicle control.

^2^The expression intensity was categorized by minimal (0 ~ 1×10^4^), mild (1×10^4^ ~ 3×10^4^), moderate (3×10^4^ ~ 5×10^4^), and strong (> 5×10^4^) based on the densitometric intensity.

All types of ZnO NPs of both sizes exerted significant decreases in the levels of coagulation factors associated with the common pathway, including factors II and V in the supernatant. The decreased levels of coagulation factors in the common pathway were consistent with the increased levels of coronated coagulation factors (factor II and V), which were observed by western blot analysis, while factor X, which is another common pathway coagulation factor, was significantly reduced in the supernatant of pristine ZnO NPs (both 20 and 100 nm).

Profiles of intrinsic pathway coagulation factors in the supernatants showed that the levels of factors IX and XII among factors VIII, IX, XI, and XII was significantly reduced by 20 and 100 nm-sized pristine ZnO NPs and 100 nm-sized citrate ZnO NPs compared to vehicle controls. The reduced pattern of factor IX and XII in the supernatant was consistent with the increased levels of coronated factor IX and XII, while factor VIII and XI showed no significant changes in the supernatant compared to vehicle control.

Profiles of extrinsic pathway coagulation factors in the supernatants revealed that levels of factor VII among factors III and VII was significantly reduced by 20 nm-sized pristine ZnO NPs and 100 nm-sized pristine and citrate ZnO NPs. The reduced pattern of factor VII in the supernatant was consistent with the increased levels of coronated factor VII, while factor III showed no significant changes in the supernatant compared to vehicle controls.

## Discussion

ZnO NPs have many advantages for use in biomedical applications such as vaccine adjuvant formulation, chemotherapy agents, and biosensors. ZnO NPs or ZnO-composites are applied for vaccine adjuvant use because the particulate form of NPs can efficiently deliver antigens to dendritic cells with subsequent adequate inflammatory responses [27, 28]. ZnO NPs have also been proposed as therapeutic agents in cancer [29–31] and inflammation [32]. However, the direct contact of ZnO NPs with blood components such as platelets can be critical in some instances, involving platelet activation and thrombus generation, but there is little information about the impact of size and surface functionalization of ZnO NPs on platelet homeostasis.

ZnO NPs were synthesized with two different sizes (20 and 100 nm) and three different functional groups (pristine, citrate, and L-serine). Since citrate and L-serine-modified ZnO NPs were originated from the pristine ZnO NPs, all types of ZnO NPs showed similar sizes and spherical shapes, but each NP type showed distinct zeta potentials due to the negatively charged citrate and positively charged L-serine moieties. FT-IR analysis confirmed that functional groups were attached to the surface of ZnO NPs. The XRD data imply that the surface modification processes did not significantly change the crystalline phase of the ZnO NPs. In this study, we found that ZnO NPs reduced the levels of coagulation factors in serum supernatant with surface functionalization-specificity. Among intrinsic coagulation factors, pristine ZnO NPs lowered the levels of factor IX and XII but not for factor VIII or XI. Likewise, pristine ZnO NPs lowered the levels of factor VII but not factor III among extrinsic coagulation factors. Interestingly, the pattern of coagulation factors in the supernatant was consistent with the adsorbed levels of coagulation factors on the NP surfaces, which might imply that ZnO NPs simply adsorb coagulation factors rather than stimulate these factors. Whether the adsorbed coagulation factors are functional or not is important for the resultant platelet homeostasis. The data for coagulation time and thrombin generation in this study imply that the adsorbed coagulation factors are not functional because the reduced levels of coagulation factors in the supernatant induced delayed coagulation time and reduced potential for thrombin generation. The selective adsorption of proteins on the surface of NPs is most likely *via* electrostatic interactions and thus the charges of target proteins and surface charges of NPs are important for binding affinity [33, 34]. There are no studies demonstrating whether coagulation factors bound to ZnO NPs were functional or not. However, there are several previous studies indicating that the adsorbed proteins can undergo conformational changes including misfolding and unfolding. For example, bovine serum albumin bound to gold NPs and tubulin protein bound to titanium dioxide NPs showed conformational changes [35, 36]. Apolipoprotein bound to gold NPs showed conformational changes (unfolding) by charge-transfer interactions [37]. Bovine α-lactoalbumin bound to silver NPs showed conformational changes, which drastically reduced the bactericidal activity of silver NPs [38]. Jack bean urease bound to silver NPs showed conformational changes and resulted loss of enzymatic activity [39].

The kinetics data of thrombin generation in this study showed that ZnO NPs reduced the thrombin generation potential with functionalization-specificity in the order pristine > citrate > L-serine, but there was no size-specificity. Consistent with the thrombin kinetics data, the profile of coagulation factors measured in the supernatants showed that pristine ZnO NPs reduced levels of intrinsic pathway coagulation factors including factors IX and XII, extrinsic pathway coagulation factors including factor VII, and common pathway coagulation factors including factor II, V, and X. The reduced levels of coagulation factors in the supernatant might be due to the adsorption of coagulation factors onto the surface of ZnO NPs because the pattern of reduced levels in the supernatant was consistent with the increased intensity of these coagulation factors observed by western blot analysis. Two functionalized 20 nm-sized ZnO NPs and L-serine-modified 100 nm-sized ZnO NPs showed high adsorption properties only for common pathway coagulation factors including factors II and V, while citrate-modified 100 nm-sized ZnO NPs showed high adsorption capacity of coagulation factors in intrinsic (factors IX and XII), extrinsic (factor VII), and common (factors II and V) pathways. Previous studies showed that some NPs such as silver NPs [40], Fe_2_O_3_ coated with polyvinyl pyridine [41], and aminated polystyrene NPs [26] inhibited intrinsic coagulation factors, while some NPs such as carboxylated and aminated multi-walled carbon nanotubes [42] and carboxylated polystyrene NPs [26] activated intrinsic or extrinsic coagulation pathways. In this regard, the profile of intrinsic, extrinsic, and common pathways shown in this study may provide important information for surface functional group-specific adsorption and inactivation of these factors.

The data involving coagulation times performed in this study showed that ZnO NPs delayed coagulation time with no functionalization- or size-specificity. Each coagulation factor could be effective on aPTT and PT but there was no remarkable difference in aPTT or PT in spite of some particles absorbing more factors than others. This result implies that the adsorption of common pathway coagulation factors could be sufficient for delaying the aPTT and PT, although the adsorption potentials are variable by types of ZnO NPs. The impact of size and functionalization of ZnO NPs on the aPTT and PT was not studied previously and the delayed aPTT and PT by other NPs were also poorly understood. Previous *in vitro* studies using carboxylated nanodiamonds, amorphous silica, and chitosan NPs showed no effect on aPTT and PT [43–45].

## Conclusion

The results found in this study indicate that ZnO NPs administered into the blood vessels can adsorb coagulation factors in a surface functional group-specific manner without activating these factors, and that the adsorbed coagulation factors are not functional since the reduced levels of coagulation factors in the supernatant were consistent with the delayed thrombin generation times and potentials. In addition, the ZnO NPs delayed coagulation time without size- or surface functional group-specificity because all types of ZnO NPs adsorbed the common pathway coagulation factors, although some types of NPs adsorbed more factors than others.

## Supporting information

S1 Fig X-ray diffraction patterns of the ZnO NPs. (A) Pristine 20 nm, (B) Citrate 20 nm, (C) L-serine 20 nm, (D) Pristine 100 nm, (E) Citrate 100 nm, and (F) L-serine 100 nm.
(PDF)
