# Supplementary material for: Surface functionalization-specific binding of coagulation factors by zinc oxide nanoparticles delays coagulation time and reduces thrombin generation potential in vitro
Source: PLoS One. 2017 Jul 19;12(7):e0181634. doi: 10.1371/journal.pone.0181634 (PMC5517067; doi:10.1371/journal.pone.0181634)
Supplement: S1 Fig — (A) Pristine 20 nm, (B) Citrate 20 nm, (C) L-serine 20 nm, (D) Pristine 100 nm, (E) Citrate 100 nm, and (F) L-serine 100 nm. (PDF) [file pone.0181634.s001.pdf]

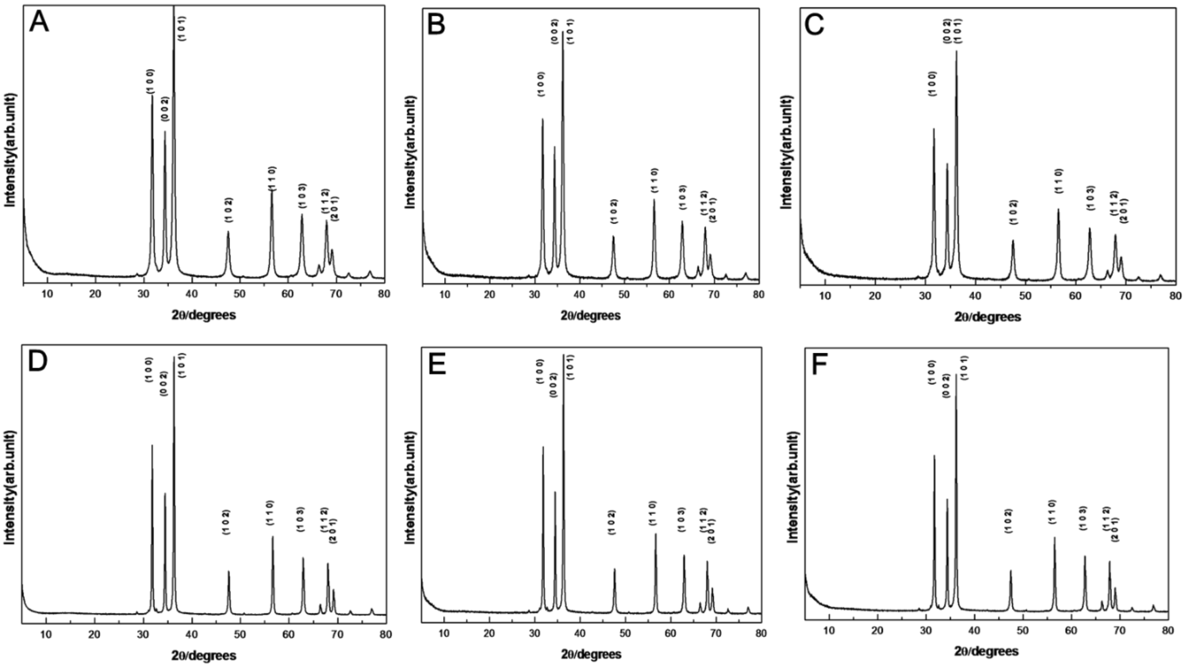

S1 Fig. X-ray diffraction patterns of the ZnO NPs. (A) Pristine 20 nm, (B) Citrate 20 nm, (C) L-serine 20 nm, (D) Pristine 100 nm, (E) Citrate 100 nm, and (F) L-serine 100 nm.
